# Some Evidence That Truth-Tellers Are More Attractive Than Liars

**DOI:** 10.1177/01461672231207567

**Published:** 2023-10-27

**Authors:** Leanne ten Brinke, Isaac Raymundo, Merusha Mukherjee, Dana R. Carney

**Affiliations:** 1 University of British Columbia – Okanagan, Kelowna, Canada; 2 Columbia University, New York City, USA; 3 University of California, Berkeley, USA

**Keywords:** attraction, deception, gender differences, nonverbal, openness, social judgment, warmth

## Abstract

Despite the prevalence of deception, people rarely doubt others’ sincerity. However, indirect evaluations of liars and truth-tellers may differ even in the absence of suspicion about veracity. Across three studies, we provide evidence for the truth attraction effect in two samples of target stimuli and three samples of participant judges. Target people are perceived as more attractive when telling the truth versus when they lie, an effect mediated by target warmth and openness. The truth attraction effect is stronger for female targets (vs. males); however, it is unaffected by the gender of the judge. Findings suggest people may be more likely to approach truth-tellers versus liars, even when not actively judging veracity. We discuss the challenges and benefits of treating both targets and participants as random factors in linear mixed-effect analyses and join the chorus of calls to increase the number of target stimuli in deception research.

Lies, if we believe them, distort our understanding of reality and can erode the foundation upon which our relationships sit (Tyler et al., 2006). Being misled can result in a loss of status, social capital, material resources, and miscarriages of justice (e.g., Kray et al., 2014; Vrij, 2000). Despite the potential costs of being deceived, people have a pervasive truth bias when asked directly to judge whether another person is lying or telling the truth. In addition, people rarely even consider the possibility they may be deceived in many different kinds of social interactions (e.g., Bond & DePaulo, 2006; Levine et al., 1999). Unsurprisingly, researchers have concluded that people are poor lie detectors. However, people may not need to actively consider veracity to discriminate between liars and truth-tellers and make wise social decisions. Theorizing by ten Brinke et al. (2016) argued that indirect social evaluations, wherein liars are perceived more negatively than truth-tellers, may reduce the likelihood of becoming ensnared in relationships with untrustworthy partners.

We report three studies (and four additional studies in the Supplemental Materials) examining whether people discriminate between liars and truth-tellers in an indirect manner: On measures of interpersonal attraction and perceived attractiveness. Because of the potential importance of these evaluations to reproduction, we initially adopted an evolutionary lens to guide our hypotheses. In the interest of theoretical and methodological transparency, we take the reader on our journey from theory to testing, and through results that changed our minds about the evolutionary framework that inspired this program of research. Nonetheless, we provide evidence of a novel effect and a mechanism that is consistent with our initial hypotheses. We begin by explaining our initial inspiration for this work below.

## “Indirect” Judgments of Liars and Truth-Tellers

Previous research has examined whether people discriminate liars from truth-tellers on various indirect measures, usually directing their attention toward a particular behavioral cue to deception. For example, Vrij et al. (2001) found that participants rated liars as “thinking harder” than truth-tellers, consistent with research showing cognitive depletion is associated with deception (Vrij et al., 2008). Similarly, DePaulo and Morris (2004) found that people rated liars as more ambivalent than truth-tellers, consistent with the emotional arousal theory of deceptive behavior (Zuckerman et al., 1981). Indeed, work by Street and Richardson (2015) suggests that these types of indirect judgments may improve on direct evaluations of veracity by directing judges’ attention to empirically valid cues to deception. Thus, not all “indirect” measures are expected to discriminate between liars and truth-tellers (Bond et al., 2015)—only those with some theoretical and empirical basis. Here, we offer our initial theoretical argument, proposed underlying mechanism, and empirical evidence for interpersonal attraction as an indirect judgment able to differentiate liars from truth-tellers.

## Evolution, Deception, and Attraction: Our Initial Theoretical Lens

First, we hypothesized that perceivers would be more attracted to truth-tellers than liars (Hypothesis 1 [H1]). This prediction was supported both by evolutionary psychology theory and empirical findings on the behavioral cues most reliably present during deception. Paternal investment theory proposes that different psychological mechanisms evolved in males and females because of the unique adaptive challenges each gender faced in their efforts to survive and reproduce (Buss, 1994). Females generally seek an honest and committed long-term partner, who is likely to contribute to parenting offspring but appear to show increased interest in more masculine and less committed males during peak fertility (i.e., ovulatory shift; Penton-Voak et al., 1999). Accordingly, some long-term male partners may be duped into investing time and resources into children that are not their genetic offspring (i.e., cuckoldry; Brase, 2006; Buss, 1994; Gangestad et al., 2005). It is estimated that female adultery occurs in 15% to 50% of all relationships and the prevalence of extra-pair paternity has been placed between 1% and 3% in Western European generations (Larmuseau et al., 2013). Given that the risk of cuckoldry is real, human males display various psychological adaptations (e.g., jealousy; Buss et al., 1992). Relatedly, we predicted that (heterosexual) males might be particularly attuned to cues to deception in potential (female) mates (Hypothesis 2 [H2]). In other words, we expected that perceivers would be more attracted to truth-tellers than lie-tellers overall but that this effect would be stronger among males rating females, relative to females rating males.

Attraction ratings were expected to be mediated by nonverbal cues to deception. Although attraction is unlike many previous “indirect” judgments that concern specific behavioral cues (e.g., “thinking hard”; Vrij et al., 2001), this judgment is informed by behaviors that may vary across liars and truth-tellers. For example, liars appear more tense, are less immediate, and are more ambivalent than truth-tellers (DePaulo et al., 2003). These same cues inform warmth and openness attributions and are associated with platonic liking, affection, affiliation, and sexual attraction (e.g., Andersen, 1985; Carney, 2021; Chartrand & Bargh, 1999; Chartrand & Lakin, 2013; Romaniuk & Terán, 2022; Vacharkulksemsuk et al., 2016). Accordingly, we hypothesized that attributions of warmth/openness would mediate the association between the veracity of targets’ statements and attraction ratings (Hypothesis 3 [H3]).

We tested these hypotheses in a total of seven studies (three appearing here, and four additional studies in the Supplemental Online Material; SOM). All instructions and measures (including those not analyzed) can be found in the Methodology file; data for all studies can be found on OSF: https://osf.io/38tbv/. All data exclusions are reported in the respective methods sections and were determined *a priori*.

## Study 1

We used a stimulus set of 24 video-recorded interrogations (13 truth-tellers and 11 lie-tellers), with content-filtered audio, of targets in a high-stakes mock-crime. Importantly, participants were *not* made aware that some speakers were lying while others told the truth. Participants indicated how attracted they were to each target, and rated targets on warmth and openness, along with other judgments to mask the objective of the study. We then examined the effect of target veracity (i.e., whether the target was lying or telling the truth) on attraction ratings (H1), moderation of this effect by target gender (H2), and mediation through perceiver attributions of target warmth and openness (H3).

## Method

### Participants

Two hundred and thirty-one participants completed the study through a cloud-based online data collection platform. Those who failed attention checks (*n* = 4), or whose videos failed to load (*n* = 6), were excluded from the analyses, leaving usable data from 221 participants. In total, these participants provided 2,652 judgments of attraction; this number far exceeds suggestions by Levine et al. (2022) that deception detection studies include >500 judgments. 112 participants identified as male and 109 as female. In all, 74.7% identified as White, 9% Black and/or African American, 6.3% Latine, 9% Asian/Pacific Islander, and 1% other/mixed-race. In the context of a paired-sample *t*-test, sensitivity analyses indicate that this sample size is sufficient to find a small (*d* = .19) effect in a test of H1, setting α at .05 and 1 − β at 80%. The age of participants ranged from 19 to 68 years old (*M* = 33.72; *SD* = 10.42); 91.4% of participants identified as heterosexual.^1^ This study was not pre-registered, but deidentified data are available at: https://osf.io/38tbv/.

### Materials: High-Stakes Mock Crime Scenario (24 Videos)

The 24 videos used in this study were created by ten Brinke et al. (2015) as part of a larger study that employed a high-stakes mock crime paradigm. Participants (i.e., targets) were randomly assigned to steal or not steal a US$100 bill from an experimental room via computerized instructions. The experimental room was also manipulated on a between-subjects basis to be enriched (i.e., brightly decorated) or sparse (i.e., no decorative objects). Regardless of the veracity assignment, all were incentivized to appear honest; participants could keep the $100 bill if the interrogator believed they were truthful. Interrogators (blind to veracity) asked a series of video-recorded questions, including two baseline (e.g., “Please describe for me, what you are wearing today, in as much detail as possible.”) and six critical questions (concerning the theft; e.g., “Did you steal the money from this office?” and “Why should I believe you?”). Salivary cortisol measures were collected pre- and post-interrogation, and verbal and nonverbal coding of participants’ behavior during the interrogations was conducted (see ten Brinke et al., 2015 for more details). Of the 81 participants in the original experiment, 63 provided consent for the use of their video in future experiments. For the purposes of this study, we used only videos of individuals in the sparse condition, as they were less visually distracting for observers and included more behavioral cues to deception than those in the enriched condition (ten Brinke et al., 2015). To select which sparse condition videos to use, still images of truth-tellers and lie-tellers holding neutral facial expressions, prior to answering any of the interrogator’s questions, were pretested for baseline physical attractiveness ratings (*N* = 40 raters). Using these ratings to balance on attractiveness, six male truth-tellers (*M* = 3.99, *SD* = 0.59), six male liars (*M* = 3.96, *SD* = 0.58) seven female truth-tellers (*M* = 4.01, *SD* = 0.46), and five female liars (*M* = 3.88, *SD* = 0.49) were selected.^2^ Videos and associated target data are available for research purposes upon request.

### Content Filtering Process: Randomly Spliced Audio

Videos were edited to include only critical questions (i.e., those relevant to the veracity manipulation). To obscure the content of the conversation, audio was spliced into 250 ms segments, randomly rearranged, and mapped back onto the video (Scherer et al., 1985). Doing so excludes words but retains paralinguistic nonverbal behaviors such as rate of speech, how much a person spoke, pitch, pauses, vocal variation, and volume. Because the nature of the questions concerned theft, deception, and naturally aroused suspicion, content filtering was necessary to ensure that participants did not infer that the study was about deception detection. This was critical (along with a number of “foil” judgments presented) to test our hypothesis that participants would indirectly discriminate between truth-tellers and lie-tellers even when participants were not cued to the possibility of deception.

### Procedure

After providing consent, participants were told they would be watching a series of videos with incomprehensible content. As a cover story, they were told that the purpose of the study was to determine how people perceive others when they cannot understand the content of what is being said. The videos were presented in a cross-gender manner such that males only observed female targets (target *n* = 12) and females only observed male targets (target *n* = 12). This number of stimuli is typical of deception detection research and almost double the median number of stimuli (*n* = 16) in studies included in Bond and DePaulo’s (2006) meta-analysis of deception detection accuracy. Videos were presented in a randomized order. After each video, participants made a series of ratings (e.g., “How relatable is this person?”) and judgments about the likely nature of the conversation (e.g., “Is this person talking to his/her significant other?”). These ratings were part of the cover story and not analyzed. All questions are listed in the Methodology file. Of primary interest, participants were asked, “How attracted are you to this person?,” and the two attributes we predicted to be potential mediators, “How warm is this person?” and “How open is this person?” All were measured on 7-point Likert-type scales, with responses ranging from 1 (*not at all*) to 7 (*very*). At the end of the survey, participants answered demographic questions.

## Results

### Were Participants More Attracted to Truth-Tellers Than Lie-Tellers?

**Figure 1.**
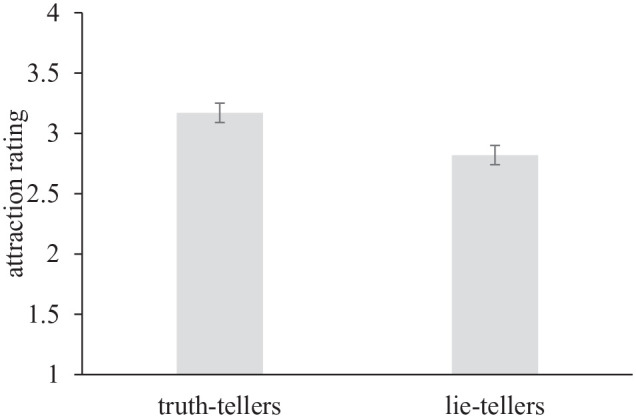
Participants Report Being More Attracted to Truth-Tellers Than Lie-Tellers. Error Bars Represent Standard Errors.

In initial analyses, participant attraction ratings were averaged across targets, such that each participant had two composite attraction ratings; one for the truth-telling targets and one for lie-telling targets they had observed. Overall, participants reported being more attracted to truth-tellers (*M* = 3.17, *SD* = 1.23) than lie-tellers (*M* = 2.82, *SD* = 1.18), *t*(220) = 7.99, *p* <.001, *d* = .53, 95% CI [0.40, 0.68], see Figure 1.

**Figure 2.**
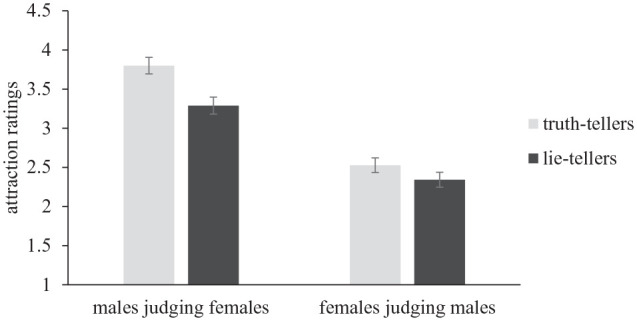
The Truth-Attraction Effect Is Stronger for Males Judging Females Than Vice-Versa. Error Bars Represent Standard Errors.

To test the hypothesis that the effect would be stronger for males judging females than vice versa (H2), we conducted a 2 (veracity: truth vs. lie) × 2 (participant gender: male vs. female) mixed analysis of variance (ANOVA). Results indicated a significant main effect of veracity, *F*(1, 219) = 67.10, *p* < .001, η^2^_p_ = .235, as reported above. In addition, there was a significant main effect of participant gender, *F*(1, 219) = 65.63, *p* < .001, η^2^_p_ = .231. Male participants were more attracted to female targets (*M* = 3.55, *SD* = 1.08) than were female participants attracted to male targets (*M* = 2.44, *SD* = 0.95). Finally, there was significant veracity by participant gender interaction, *F*(1, 219) = 14.69, *p* < .001, η^2^_p_ = .063. Specifically, males were more attracted to female truth-tellers (*M* = 3.79, *SD* = 1.12) than female lie-tellers (*M* = 3.29, *SD* = 1.15), *t*(111) = 7.62, *p* < . 001, *d* = .72, 95% CI [0.51, 0.93]. Females also perceived male truth-tellers (*M* = 2.53, *SD* = 0.98) to be more attractive than male lie-tellers (*M* = 2.34, *SD* = 1.00), *t*(108)= 3.59, *p* < .001, *d* = .34, although this effect was somewhat weaker (see Figure 2).

### Did Warmth and Openness Mediate the Truth Attraction Effect?

To test if warmth mediated the effect of target veracity on attraction ratings, we used the MEMORE analysis for SPSS to run a within-subjects bootstrap mediation with 5,000 simulations (Montoya & Hayes, 2017). Attraction ratings for truth-tellers and lie-tellers were the dependent variables, and warmth ratings of truth-tellers and lie-tellers were the mediating variables. Ratings of target warmth mediated the relation between attractiveness judgments and veracity of target (indirect effect = .25, *SE* = 0.05, 95% CI [0.16, 0.35]). Using the same approach, we tested the mediating role of openness, which also mediated the relation between attractiveness judgments and veracity of target (indirect effect = .19, *SE* = 0.04, 95% CI [0.11, 0.27]).

### A Mixed Model Approach

While many researchers still provide ANOVA-based analyses of deception detection tasks, it is becoming more common in judgment studies involving multiple stimuli to report mixed models; thus, we also conducted linear mixed model analyses which add a random effect of the target to the analyses. This has the benefit of reducing the Type I error rate by treating stimuli as a sample of liars and truth-tellers, much like participants are a sample of the broader population (Judd et al., 2012). Treating stimuli as a random effect also provides some confidence that findings are likely to generalize to other sets of stimuli.

To test whether veracity predicted attraction ratings we ran a multilevel model with veracity as the predictor, participant ID and target ID as random effects, and attraction ratings as the outcome variable. Reported *t*-tests use Satterthwaite’s method. Contrary to our predictions [H1], we did not find a significant effect of veracity on attraction, *b* = .21, 95% CI [−0.12, 0.55], *t*(2647) = 1.25, *p* = .23, such that participants were not more attracted to truth-tellers (*M* = 3.21, *SE* = 0.24) than lie-tellers (*M* = 2.78, *SE* = 0.26),^3^ although the means were in the expected direction.

Next, we tested whether the effect was moderated by the participant’s gender (H2). Specifically, we ran a multilevel model with veracity, gender, and their interaction terms as predictors, participant ID and target ID as random effects, and attraction ratings as the outcome variable. Veracity and gender variables were both effect-coded (−1, 1) to aid interpretation. There was no significant main effect of veracity, *b* = .17, 95% CI [−0.10, 0.45], *t*(2645) = 1.23, *p* = .22, but there was a significant main effect of gender, *b* = .55, 95% CI [0.25, 0.86], *t*(2645) = 3.58, *p* < .001. Male participants were significantly more attracted to female targets (*M* = 3.54, *SE* = 0.22) than female participants were to male targets (*M* = 2.44, *SE* = 0.22). Critically for H2, however, there was no significant interaction between veracity and participant’s gender, *b* = .08, 95% CI [−0.19, 0.36], *t*(2645) = 0.58, *p* = .57.

Finally, we tested for an indirect effect of veracity on attractiveness through perceived warmth and openness separately using the Z_Mediation_ statistic (Fiedler et al., 2018; Iacobucci, 2012).^4^ Contrary to H3, we found that perceived warmth did not mediate the relationship between veracity and perceived attractiveness, Z_Mediation_ = 1.85, 95% CI [−0.11, 3.81], *p* = .06. Likewise, we found that openness did not mediate the effect of veracity on attractiveness, Z_Mediation_ = 1.84, 95% CI [−0.12, 3.80], *p* = .07.

### Analysis Summary

Using traditional ANOVA approaches to analyzing our data, we found support for all hypotheses such that truth-tellers were perceived as more attractive than liars (H1), that this effect was stronger for male participants judging female targets than vice versa (H2), and that this effect was mediated by perceptions of warmth and openness (H3). However, these findings did not replicate in mixed models that account for variability in targets suggesting that this effect may not generalize beyond this particular set of stimuli. Similarly, a series of four studies appear in the Supplemental Online Material which use similar designs and sets of targets. In these studies too, ANOVA approaches provide support for hypotheses, but effects do not replicate in mixed model analyses of the same data.

## Study 2

Here, we set out to design a higher-powered design and more direct control condition to test our hypotheses. Specifically, Study 1 attempted to match targets in the truth-telling and lie conditions on physical attractiveness, Study 2 provided a more rigorous control condition by asking participants to rate target stimuli either (a) during critical questions on which veracity was manipulated or (b) during baseline questions from the same interview in which all participants told the truth. This design also had the effect of doubling the number of target stimuli included in the analyses. Increasing the number of target stimuli is advised in linear mixed effect models, and has been identified by Levine et al. (2022) as more important for producing replicable findings than increasing the number of participants providing ratings. Accordingly, this study used the same targets as Study 1 but added 24 new videos of responses to baseline questions from each target individual. Evidence for the truth attraction effect was expected in the form of veracity by video-type interaction (Hypothesis 4 [H4]). Despite inconsistent results across statistical approaches, we continued to test whether the truth attraction effect was moderated by gender [H2], and mediated by impressions of target warmth and openness [H3].

## Method

### Participants

Two hundred and one paid subjects completed the study through a West Coast paid participant pool. Participants were excluded due to failing attention checks (*n* = 28), or reporting their videos did not have sound and/or visuals (*n* = 19),^5^ leaving data from 162 participants. In total, these participants provided 1944 attraction judgments. 60 identified as male, 101 as female, and 1 as other.^6^ In all, 22.8% identified as White, 6.8% Latine, 63.6% Asian/Pacific Islander, and 6.8% identified as other/mixed race. Ages ranged from 18 to 53 years old (*M*_age_ = 21.09, *SD* = 3.03); 90.7% identified as heterosexual.^7^ This study was not pre-registered, but deidentified data are available at: https://osf.io/38tbv/

### Materials

Targets were the same as those featured in Studies 1 to 2 (*N* = 24). However, this study included the addition of baseline question videos for each target (*n* = 24) in addition to the critical question videos (*n* = 24, as used in Studies 1 to 2) for a total of 48 videos. In baseline question videos, targets answered questions unrelated to the theft that were verifiably true (e.g., “Please describe for me, what you are wearing today, in as much detail as possible.”), before they responded to critical questions about stealing the $100 (e.g., “Did you steal the money from this office?”). As in previous studies, participant judges were not informed that some videos were truths or lies and that all videos were content-filtered. Again, this was necessary to ensure that participants did not infer that the study was about deception detection and to test whether attraction ratings would discriminate liars versus truth-tellers when participants were not cued to the possibility of deception.

### Procedure

The procedure was the same as in Study 1, with the only difference being that participants were randomly shown either the critical question video, in which veracity was manipulated (i.e., the truth/lie manipulation moment), or the baseline video, in which all targets were honest (i.e., before the truth/lie manipulation moment), for each of 12 targets. Participants watched opposite gender targets (i.e., males rated females and vice versa), and responded to the same attraction, veracity, and warmth/openness outcome variables as in Studies 1 to 2. Participants then answered demographic questions. The exact wording of these and all distractor questions can be found in the associated Methodology file.

## Results

**Figure 3:**
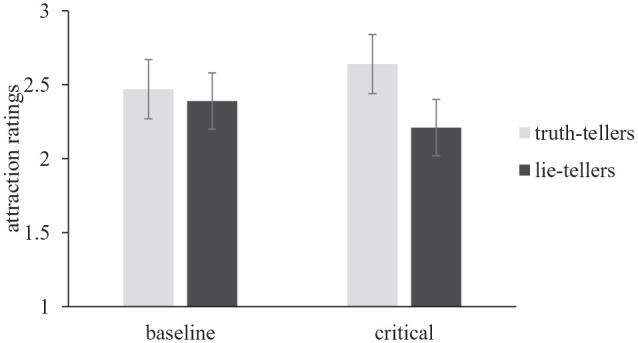
Interaction Between Video Type (Baseline vs. Critical Truth/Lie Question) and Target Veracity on Attraction Ratings. Error Bars Represent Standard Errors.

Recognizing that linear mixed models provided a more conservative test of hypotheses in Study 1, we elected to take this approach to the analysis of Study 2 data. Specifically, to examine whether the relation between veracity and attraction was moderated by the type of video participants watched [H4], either a baseline video in which all targets told the truth or a critical video in which half the targets lied, we ran a multilevel model with veracity, video type, and their interaction term as the predictors, participant ID and target ID as random effects, and attraction ratings as the outcome variable. Predictors were effect-coded (−1, 1) to aid interpretation. Reported *t*-tests use Satterthwaite’s method. We found no significant main effect of veracity, *b* = .13, 95% CI [−0.12, 0.37], *t*(1937) = 1.01, *p* = .33, nor a significant main effect of video type, *b* = −.004, 95% CI [−0.05, 0.04], *t*(1937) = .05, *p* = .86. However, as predicted in H4 we found a significant interaction between veracity and video type, *b* = .09, 95% CI [0.04, 0.13], *t*(1937) = 3.56, *p* < .001 (see Figure 3). Simple effects analyses revealed that participants reported being less attracted to lie-tellers during their critical video (*M* = 2.21, *SE* = 0.20), relative to their baseline video (*M* = 2.39, *SE* = 0.20), *t*(1794) = 2.56, *p* = .011. By contrast, participants were more attracted to truth-tellers during their critical video (*M* = 2.64, *SE* = 0.19), relative to their baseline video (*M* = 2.47, *SE* = 0.19), *t*(1793) = −2.48, *p* = .013.

Next, we examined whether gender moderated this interaction [H2]. To test this, we ran a multilevel model with veracity, video type, gender, and their interaction terms as predictors, participant ID and target ID as random effects, and attraction ratings as the outcome variable. Again, predictors were effect-coded (−1, 1) to aid interpretation. We found no significant main effects of veracity, *b* = .12, 95% CI [−0.13, 0.38], *t*(1933) = 0.97, *p* = .35, video type, *b* = .001, 95% CI [−0.05, 0.05], *t*(1933) = 0.06, *p* = .97, nor gender, *b* = .04, 95% CI [−0.24, 0.33], *t*(1933) = 0.36, *p* = .72. Contrary to H2, we found no significant three-way interaction between veracity, video type, and gender, *b* = −.04, 95% CI [−0.08, 0.01], *t*(1933) = −1.40, *p* = .16, but the interaction between veracity and video type remained significant in the model, *b* = .08, 95% CI [0.03, 0.13], *t*(1933) = 3.08, *p* = .002.

Finally, we tested for indirect veracity on attraction ratings through perceived warmth and openness separately (H3). Here, we focus our mediation analysis only on the critical videos. Contrary to H3, we found that perceived warmth did not mediate the effect of veracity on attraction for the critical videos, Z_Mediation_ = 1.69, 95% CI [−0.26, 3.65], *p* = .09. Similarly, we found that perceived openness did not mediate the relationship between veracity and attraction for the critical videos, Z_Mediation_ = 1.79, 95% CI [−0.16, 3.75], *p* = .07.

Study 2 provided evidence for the truth attraction effect, such that participants were less attracted to lie-tellers during critical questions relative to baseline questions (i.e., when the same target individuals were telling the truth). Interestingly, truth-tellers showed the opposite pattern; participants were more attracted to truth-tellers during critical questions relative to baseline questions. This is consistent with previous research by ten Brinke et al. (2015) suggesting that truth-tellers become less stressed (as indexed by cortisol reactivity) over time as they answer baseline followed by critical questions, whereas liars become more stressed. Importantly, the inclusion of a random effect of stimuli in the model suggests that these findings are likely to generalize to other truth and lie statements. However, we did not find that gender moderated this effect, suggesting that this effect is not strongly rooted in evolutionary theories of parental investment (Buss, 1994; also see Study 1 in Supplemental Online Materials). In addition, we did not find that the truth attraction effect was mediated by perceptions of warmth and openness. However, mediation analyses approached statistical significance in Study 2 using only critical video questions and were statistically significant using an ANOVA approach in Study 1 with the same stimuli. Together, these effects suggest that we may be underpowered to find significant mediation effects in a mixed-model context. As such, we believe that additional testing of our hypotheses is warranted in a study with a greater number of target stimuli.

## Study 3

The purpose of Study 3 was to test the generalizability of the truth attraction effect. First, we shifted to a more realistic context in which participants could both see and hear the content present in a plea, monologue, or interaction. We also wanted to use a novel set of stimuli and adopted the Miami University Deception Detection Database (MU3D; Lloyd et al., 2019) for this purpose. The MU3D includes Black and White men and women telling lies and truths about people they do and do not like. The MU3D has the benefit of including many targets while presenting content that does not immediately arouse suspicion about deception. We also shifted to study ratings of attractiveness (vs. attraction). Attractiveness is assessed quickly and reliably by others (Willis & Todorov, 2006), and does not imply sexual attraction. Specifically, we had participants make attractiveness ratings of both target men and women, allowing us to examine whether the truth attraction effect was specific to cross-gender judgments, or not. Despite these changes to the design, we tested the same hypotheses. Specifically, we examined the effect of target veracity on attractiveness ratings (H1), moderation of this effect by target gender (H2), and mediation through perceiver attributions of target warmth and openness (H3).

### Participants

Undergraduate students were recruited using the undergraduate participant pool at a Western Canadian university. All students at least 18 years of age seeking course credit on SONA were eligible to participate. A total of *N* = 290 participants (183 identified as women, 70 identified as men, and 5 as non-binary) engaged in the study. Of these, 210 (72.4%) identified as heterosexual, 32 as bisexual, 7 as asexual, 4 as pansexual, 4 as queer, 2 as gay, 2 as lesbian, 2 as another category not listed, and 4 selected “prefer not to answer.” Note that participants had the option to select multiple gender identities and sexualities or not complete these questions; accordingly, responses here do not sum to the total number of participants. In total, these participants provided 5080 attractiveness judgments. No data exclusions were made. The mean age of our receivers was 20.38 (*SD* = 3.17).

### Materials

Materials included 80 videos of senders describing their interpersonal relationships, taken from the MU3D (Lloyd et al., 2019). This database includes 80 senders (40 females and 40 males) with a mean age of 20.13 (*SD* = 1.56), each producing four low-stakes lie detection videos, which crossed valence (positive vs. negative) with veracity (truth vs. lie). For each video, the experimenter gave the sender a prompt, left the web-cam-equipped cubicle, then returned 45 s later and stopped the recording. Senders were instructed to talk freely about (a) a person they genuinely liked, (b) a person they genuinely disliked, (c) the person they liked as if they disliked that person, and finally (d) the person they disliked as if they liked that person.

For the purposes of the current study, only positively valence videos were selected and, to reduce potential sources of variability, only White individuals were included as target stimuli. As such, this study included 80 stimuli videos—40 targets (20 White males, 20 White females) each providing one positive truth and one positive lie. Receivers watched a randomized subset of 20 stimuli including 20 different target individuals (5 truth-telling females, 5 truth-telling males, 5 lie-telling females, and 5 lie-telling males). Four subsets were created. Participants were randomly assigned to view one subset; stimuli within each subset were presented in random order.

### Procedure

Participants were randomly assigned to watch one subset of 20 videos from the MU3D (Lloyd et al., 2019), in which senders claimed to like an individual. Half the videos included truth-tellers (i.e., they genuinely liked the person they claimed to like), while the other half were lying (i.e., the sender did not actually like the person they claimed to like). Critically, participants were not made aware of the possibility of deception. After watching each video, receivers were asked to rate how attractive, warm, and open they perceived the target to be on 1 (*not at all*) to 7 (*very*) scales.

At the end of the study, receivers were asked to provide general demographic information (i.e., age, gender, sex, race) and to report on what they thought the study was about. They were also asked, “Did you notice anything suspicious or unusual about the videos you watched?” in an open-ended format. Only nine individuals (3.1%) indicated that some targets may have been lying or not genuine. However, a direct follow-up question asked, “At any point while watching the videos did you think someone in the videos might have been lying?”: 184 (63.4%) participants responded “yes,” 73 (25.2%) responded “no,” and 33 (11.4%) participants did not answer. Finally, receivers were fully debriefed and thanked for their time, and SONA credit was granted automatically.

## Results

To test whether veracity predicted perceived attractiveness [H1], we ran a multilevel model with veracity as the predictor, participant ID and target ID as random effects, and attractiveness as the outcome variable. As predicted, we found that veracity significantly predicted perceived attractiveness, *b* = .05, 95% CI [0.02, 0.08], *t*(5075) = 3.59, *p* < .001, such that truth-tellers (*M* = 3.89, *SE* = 0.11) were perceived as more attractive than lie tellers (*M* = 3.78, *SE* = 0.11).

Next, we examined whether the participant’s gender and the target’s gender moderated the effect of veracity on attractiveness [H2]. To test this, we ran a multilevel model with veracity, participant gender, target gender, and their interaction terms as predictors, participant ID and target ID as random effects, and attractiveness as the outcome. We found a significant main effect of veracity, *b* = .05, 95% CI [0.02, 0.08], *t*(4989) = 2.98, *p* = .003, and a significant main effect of target gender, *b* = .27, 95% CI [0.11, 0.42], *t*(4989) = 3.43, *p* = .002, but no main effect of participant gender, *b* = −.07, 95% CI [−0.21, 0.07], *t*(4989) = −1.01, *p* = .31. We also did not find a significant interaction between veracity, participant gender, and target gender, *b* = −.01, 95% CI [−0.05, 0.02], *t*(4989) = −0.75, *p* = .45. However, there was a marginal interaction between veracity and target gender, *b* = .03, 95% CI [0.00, 0.07], *t*(4989) = 1.92, *p* = .055. Consistent with H2, simple effects analyses revealed that female targets were rated as less attractive when telling a lie (*M* = 4.01, *SE* = 0.13) versus the truth (*M* = 4.17, *SE* = 0.13), *t*(4707) = −3.46, *p* < .001. By contrast, male targets did not differ in perceived attractiveness when telling a lie (*M* = 3.54, *SE* = 0.13) versus the truth (*M* = 3.57, *SE* = 0.13), *t*(4707) = −0.75, *p* = .452.

Finally, we tested for the indirect effects of veracity on attractiveness through warmth and openness, separately, via mediation. As predicted in H3, we found that warmth significantly mediated the effect of veracity on attractiveness, Z_Mediation_ = 5.97, 95% CI [4.01, 7.93], *p* < .001. Similarly, openness also mediated the effect of veracity on attractiveness, Z_Mediation_ = 3.81, 95% CI [1.85, 5.77], *p* < .001. Taken together, the results of Study 3 are consistent with H1, H2 and to H3 as well as the outcome of ANOVA approaches to hypothesis testing in Study 1.

## General Discussion

The results in this report provide evidence of a truth attraction effect, demonstrating a novel phenomenon of potential theoretical and methodological importance. Specifically, we find that—even when participants are not made aware of the possibility that some targets will be lying—ratings of attraction differentiated liars from truth-tellers. Specifically, participants were more attracted to truth-tellers than liars (H1). In Study 2, participants were less attracted to deceptive targets during the truth/lie moment (i.e., during critical questions) versus during baseline questions in which all targets were honest. Similarly, Study 3 included targets that told both truth and lies, finding a significant effect of veracity such that targets were perceived as more attractive when telling the truth than when lying about the same topic.

We also found some evidence that the truth attraction effect was stronger for female targets than male targets (H2). In Study 2, where participants only watched and provided ratings on opposite-gender targets, gender did not moderate the effect of veracity on attraction judgments. However, in Study 3, a marginal interaction between target gender and veracity (*p* = .055) emerged. Consistent with our H2 prediction, women targets were perceived as more attractive when telling the truth versus lying, whereas there was no effect of veracity on the attractiveness ratings of target men. Finally, we found—in the relatively high-powered Study 3—that perceptions of target warmth and openness mediated the relationship between target veracity and attractiveness ratings, providing a potential mechanism for the truth attraction effect [H3].

### Theoretical Implications

These data suggest that even when participants are uninformed about the veracity manipulation and methodological efforts are taken to avoid arousing suspicion, evaluations of attraction may allow people to make wise social choices—approaching truth-tellers and avoiding liars (ten Brinke et al., 2016). We found some evidence that this effect was stronger for female targets than male targets. Although this was consistent with our initial evolutionary argument, stemming from parental investment theory (Buss, 1994), our confidence in this explanation was tempered by the lack of any three-way interaction between veracity, target gender, and participant gender in Study 3. In other words, the truth attraction effect was not specific to cross-gender judgments in a predominantly heterosexual sample. Accordingly, we do not believe that these findings are best explained by evolutionary pressures. While it could be argued that we did not have the best tests of this theory (and future research may be warranted), a more parsimonious explanation for the observed gender differences may come from (a) gender differences in warmth/openness as cues to deception, and/or (b) the extent to which these cues are relevant to the perceived attractiveness of females (vs. males).

### Methodological Implication

This set of studies—including those presented in the Supplementary Online Material—provide several methodological implications. First, they illustrate how aggregating across stimuli versus treating stimuli as a random factor in analyses can result in different conclusions. Our experience in Study 1 such that ANOVA analyses provided support for hypotheses but did not replicate in linear mixed model analyses, is consistent with the warnings of Judd et al. (2012) that aggregating judgments can increase the potential of Type I errors. This is not to say, however, that studies using an aggregated data, ANOVA approach to data analysis necessarily produce false positives. Indeed, support for hypotheses reported in Study 1 ANOVAs was subsequently replicated in linear mixed effect models when more powerful study designs were employed. The MU3D (Lloyd et al., 2019) provided a critical resource for conducting a high-powered study of lie detection in a mixed-model context, given the number of stimuli included. If we are to adopt a linear mixed approach to future deception detection research, the creation of additional, large, freely available datasets of truth and lie video stimuli should be a priority for the field moving forward.

### Limitations and Future Directions

Future research on the truth attraction effect should consider using a more diverse sample of participants and creating stimuli more directly relevant to romantic relationships (e.g., dating profile videos in which targets lie or tell the truth about their identity or interests). Using actual dating profiles in which users admit to engaging in self-serving, presentational lies (e.g., Toma et al., 2008), and studying face-to-face interactions such as speed dating events (Kerr et al., 2020) will be important to understanding the generalizability and real-world implications of the truth attraction effect.

Although we provide evidence that perceptions of warmth and openness may serve as mediators of this effect, this does not preclude the existence of other potential mechanisms (Fiedler et al., 2018). Indeed, the reader may wonder precisely which micro-level verbal or nonverbal behaviors underlie the molar-level impressions of warmth, openness, and attraction studied here. For example, research suggests that people interpret immediacy cues such as smiles, nods, and eye contact as indicators of interpersonal warmth (Gifford, 1994; Kraft-Todd et al., 2017). These behaviors may also serve as mediators of the truth attraction effect. Future research should consider alternative mediators and the extent to which these micro-level behaviors or molar-level impressions replicate across contexts.

## Conclusion

The presence of deception likely shapes our behaviors and social interactions, even when we are totally unaware of its presence. We provide evidence that people are more attracted to truth-tellers, relative to lie-tellers, that this effect appears to be stronger for female targets (versus males), and that impressions of warmth and openness may be a mechanism through which these effects occur—acting uncooperative, unpleasant, and providing little detail, have been identified as behavioral predictors of deception (DePaulo et al., 2003). Given that attraction and attractiveness shape our interpersonal interactions, people may be motivated to approach truth-tellers more than lie-tellers, providing some protection from the costs of deception in our daily lives. Future research should take this phenomenon into the field and consider the implications of honesty (versus deception) on attraction and approach behaviors in real, live social interactions.

## Supplemental Material

sj-docx-1-psp-10.1177_01461672231207567 – Supplemental material for Some Evidence That Truth-Tellers Are More Attractive Than Liars Supplemental material, sj-docx-1-psp-10.1177_01461672231207567 for Some Evidence That Truth-Tellers Are More Attractive Than Liars by Leanne ten Brinke, Isaac Raymundo, Merusha Mukherjee and Dana R. Carney in Personality and Social Psychology Bulletin

## Notes

1. Although sexual orientation was not considered for exclusion/inclusion a priori, it is worth noting that all findings reported here replicate when the sample is restricted to participants identifying as heterosexual.

2. Due to experimenter error, the number of female truth-tellers and liars was slightly imbalanced. This was corrected in Study 4 of the Supplemental Online Material.

3. Means presented here are estimated means adjusted for the random effects included in the model.

4. Given that there is no standard test for an indirect effect with mixed effect models via bootstrap mediation, we leveraged a formula which calculates the Z_Mediation_ statistic (Iacobucci, 2012). This formula takes the product of the *a* and *b* coefficients divided by their standard errors respectively, then divided by their pooled standard error producing a Z_Mediation_ statistic which can be used in a *z* test to test for significance of an indirect effect.

5. *n* = 8 participants both failed attention checks and reported technical issues.

6. Only participants identifying as either male or female were used in analyses including gender as a variable.

7. All findings reported below replicate when sample is restricted to those who identify as heterosexual.
