# Supplementary material for: Some Evidence That Truth-Tellers Are More Attractive Than Liars
Source: Pers Soc Psychol Bull. 2023 Oct 27;51(6):900–9. doi: 10.1177/01461672231207567 (PMC12044204; doi:10.1177/01461672231207567)
Supplement: sj-docx-1-psp-10.1177_01461672231207567 – Supplemental material for Some Evidence That Truth-Tellers Are More Attractive Than Liars [file sj-docx-1-psp-10.1177_01461672231207567.docx]

**Supplementary Online Materials (SOM)**

**The Truth Attraction Effect: Truth-Tellers are More Attractive than Liars**

**Supplemental Studies – Some Caveats**

These studies were conducted before we adopted a linear mixed model approach to analyzing data, and include largely the same set of target stimuli as included in Study 1 of the main manuscript. Below, we present ANOVAs, which aggregate across target stimuli, and largely support hypotheses. However, linear mixed effect models do not replicate these effects. This is likely due to the small number of target stimuli involved in these studies and future research that addresses this limitation may result in consistent findings across both ANOVA and linear mixed model approaches.

**Supplemental Study 1**

The purpose of Supplemental Study 1 was to replicate findings from Study 1 (in the manuscript) and to test a potential evolutionary mechanism for the truth attraction effect. Consistent with our initial evolutionary theorizing (e.g., Buss, 1994), we tested whether the truth attraction effect would be moderated by perceived fertility. If a paternal investment mechanism was at play, we would expect that the truth attraction effect would be replicated for individuals perceived as ‘fertile’, but not for those perceived as unable to produce offspring [H5]. Using the same stimuli set as in Study 1, we tested whether the truth attraction effect would be greater for targets perceived as highly (vs. not) fertile.

**Methods**

**Participants**

Three hundred and forty individuals participated: some from a cloud-based online sample (*n* = 195) and some from a West Coast University paid participants pool (*n* = 145). Those who failed attention checks (*n* = 53), reported that the videos did not load properly (i.e., they could not see or hear the video; *n* = 37), or did not finish the survey (*n* = 5) were excluded from analyses, leaving useable data from two hundred and forty-five participants (51.4% identified as female). Sensitivity analyses suggest that this sample size is sufficient to find a small (*d* = .18) effect in a test of H5, setting α at .05 and 1 - β at 80% in an ANOVA approach to analysis. Participants identified as: 45.7% White, 8.6% Black and/or African American, 7.3% Latine, 34.3% Asian/Pacific Islander, 4.1% other/mixed-race. 119 identified as male and 126 as female. The age ranged from 18 to 93 years old (*M*age = 24.34; *SD* = 6.03); 93.9% of participants identified as heterosexual.^^[[1]](#footnote-1)^^ This study was not pre-registered, but deidentified data are available at: <https://osf.io/38tbv/?view_only=017a3e032b6e4d74889d282002b04733>

**Materials**

The same stimuli, paradigm, and dependent measures were used from Study 1. The only difference was the manipulation of perceived fertility.

**Procedure**

Before participants began observing the videos, participants were randomly assigned one of two conditions: targets were identified as “fertile” or “infertile.” In the fertile [infertile] condition, participants were given the following prompt.^^[[2]](#footnote-2)^^

*It is important to note that these people, whom you are about to watch on video, are HIGHLY FERTILE [NOT FERTILE]. In other words, through testing and biomarker analysis, they were determined to have a high [no] likelihood of fast and successful reproductive success. They were selected to be interviewed not only because they were single and looking for a romantic partner, but also because they have a very high [low] reproductive potential.*

*Various health assessments and questionnaires were performed by these individuals, and only the most [least] fertile people were selected for the videos you are about to see. So, it is important to keep that in mind while making ratings about these people (which you will start doing in a minute) -- that these people not only want a romantic partner but are highly fertile [infertile] and able [unable] to have children easily and successfully.*

After this manipulation, the procedure was the exact same as in Study 1 (from the manuscript) and participants answered demographic questions.

**Results**

We used the same procedure in Study 1 to aggregate attractiveness ratings for truth-tellers separately from lie-tellers. Consistent with H1, we found participants reported being more attracted to truth-tellers (*M* = 2.83, *SD* = .99) than lie-tellers (*M* = 2.67*, SD* = .97*), t*(244) = 4.17, *p* <.001, *d* = .27, 95% CI [.14, .39].

Using a 2 (veracity) x 2 (participant gender) x 2 (fertility manipulation) mixed ANOVA, we found a significant main effect of veracity, *F*(1, 217) = 20.80, *p* < .001, η^2^_p_ = .087, as described above. Additionally, there was a significant main effect of gender, *F*(1, 217) = 13.13, *p* < .001, η^2^_p_ = .057. Male participants were significantly more attracted to females (*M* = 2.95, *SD* = .99) than females were to males (*M* = 2.57, *SD* = .85). Contrary to H2, the veracity by gender interaction did not reach statistical significance, *F*(1, 217) = 3.32, *p* =.070, η^2^_p_ = .015. No other main effects or interactions were significant, *p*s > .344. Thus, there was no evidence that the fertility manipulation had any main effect, nor did it interact with veracity. Accordingly, we did not find evidence to support H5.

Consistent with H3, we also found significant indirect effects of target warmth (indirect effect = .19, *SE* = .04, 95% CI [.11, .28]) and openness (indirect effect = .16, *SE* = .04, 95% CI [.09, .24]).

**Analysis Summary**

Using an ANOVA approach and aggregating across target stimuli, we replicated the primary [H1], and mediational findings [H3] reported in Study 1; though, we did not find evidence to suggest that perceived fertility plays a role in the truth attraction effect [H5]. Granted, our manipulation of fertility was not without limitations. Although blunt, it may not have had the intended effect because such a manipulation does not have an analogue in real life. As such, subtler manipulations of reproductive viability—age, health, interest in children—may be more effective ways to test the proposed evolutionary origins of the truth attraction effect.

**Supplemental Study 2**

The purpose of Supplemental Study 2 was to test the generalizability of the truth attraction effect. We shifted to a more realistic context in which participants could both see and hear the target stimuli content. We also shifted to study ratings of attractiveness (vs. attraction) and asked participants to rate both same- and other-gender targets. Additionally, participants were instructed that some targets were lying while others were telling the truth, as in most previous deception detection research (Bond & DePaulo, 2006). This allowed us to assess whether the previously observed effect would generalize to a situation in which suspicion about deception was deliberately aroused, and whether the same pattern of findings would be present in a direct veracity evaluation. To match measures on sensitivity, both attractiveness (i.e., our indirect measure) and direct veracity judgments were made on binary and 7-point scales. This study also balanced the number of stimuli in each condition; the final stimulus set contained 6 female and 6 male truth-telling videos and 6 female and 6 male lying videos balanced on physical attractiveness (*M_truth_* = 3.89, *SD* = 0.91; *M_lie_* = 3.89, *SD* = 0.80, respectively). One final improvement to this study was that we used multi-item scales of warmth and openness taken from Vacharkulksemsuk et al. (2016).

**Methods**

**Participants**

Two hundred and fifty-one participants completed the study through a cloud-based participant recruitment pool using two separate postings: one for males and one for females, to recruit an equal number of each gender. This sample size was determined a priori, using a sample size calculation, and was pre-registered. As per our pre-registration, participants who failed more than two of four attention check questions presented during the video rating task would be excluded; however, all participants met attention check criteria and were retained for analysis. 126 identified as male, 124 as female and 1 as other.^^[[3]](#footnote-3)^^ 76.1% identified as White, 7.6% Black and/or African American, 5.3% Latine, 7.6% Asian, .4% South Asian, .4% Middle Eastern, and 2.8% identified as other/mixed-race. The age of participants ranged from 18 to 77 years old (*Mage* = 40.03; *SD* = 12.34*)*; 90% of participants identified as heterosexual. Study 4 was pre-registered: <https://osf.io/ztpvr/?view_only=9701a2b3f0f44d5a9be9b05d31647db2> and deidentified data are available at: <https://osf.io/38tbv/?view_only=017a3e032b6e4d74889d282002b04733>

**Materials**

The same lie/truth stimuli used in Study 1 of the manuscript were used here except for three videos which were replaced to balance the number of truth tellers and liars in the female videos while maintaining matched still-image attractiveness ratings across gender and lie/truth condition. The videos used in this study contained the full original audio (vs content filtered audio used in Study 1) and participants observed all 24 videos. That is, participants watched videos of both male and female targets (unlike in Study 1 which included cross-gender stimuli only).

**Procedures**

Unlike Study 1 where participants were told to make ratings of targets, in this study the instructions were explicit. Participants were told exactly what they would be observing: videos of people who deny stealing $100 (half of whom actually did) and that they would be answering questions about each video. Participants observed all 24 videos in a randomized order. After each video, participants rated each target on a number of traits: (1) a continuous physical attractiveness rating (on a scale from 1 to 7, “how attractive do you think this person is (as compared to all the people you have met in your life—meaning, how attractive are they as a human—when comparing to others?”), (2) a binary physical attractiveness rating (“Is this person unattractive or attractive?”), (3) a continuous deception rating (on a scale from 1 to 7, “Please rate how much you think the person in the video is lying versus telling the truth”), and (4) a binary deception rating (“Is this person lying or telling the truth?”). For each video, participants also completed a (5) 5-item warmth measure (on a scale from 1 to 7, “Please rate how much you believe the word [caring, sincere, tolerant, likeable, good natured] applies to the person in the video you watched”), and an (6) 8-item openness measure (on a scale from 1 to 7, “Please rate how much you agree with the following statement: the person in the video [is original; comes up with new ideas; is curious about many different things; is ingenuous; is a deep thinker; has an active imagination; is inventive; values artistic, aesthetic experiences; prefers work that is routine; likes to reflect; plays with ideas”]. Warmth and openness items were taken from Vacharkulksemsuk et al (2016). Participants then answered demographic questions.

**Results**

**Indirect, Attractiveness Judgments**

**Continuous Measure of Attractiveness.** We calculated average (continuous) attractiveness ratings for truth-tellers and lie-tellers. Consistent with H1, overall (across gender of perceiver and target), truth-tellers (*M* = 4.45, *SD* = 0.76) were rated as more attractive than lie-tellers (*M* = 4.30, *SD* = 0.75), *t*(250) = 6.04, *p* <.001, *d* = .38, 95% CI [.25, .51].

A 2 (veracity) x 2 (participant gender) x 2 (target gender) mixed ANOVA revealed a main effect of veracity, *F*(1, 248) = 37.41, *p* < .001, η^2^_p_ = .131, as described above. Importantly, there was also a significant interaction between veracity and target gender, *F*(1, 248) = 12.34, *p* < .001, η^2^_p_ = .047. Consistent with H2, participants perceived female truth-tellers to be more attractive (*M* = 4.56, *SD* = 0.81) than female lie-tellers (*M* = 4.32, *SD* = .81), *t*(249) = 6.54, *p* < .001, *d* = 0.41, 95% CI [28, .54]*.* Participants also perceived male truth-tellers to be more attractive (*M* = 4.35, *SD* = 0.90) than male lie-tellers (*M* = 4.29, *SD* = .90), *t*(249) = 1.94, *p* = .054, *d* = 0.12, 95% CI [.00, .25], although this effect was weaker than that observed for female targets*.* Less relevant to the hypotheses, there was also a significant main effect of target gender, *F*(1, 248) = 7.45, *p* = .007, η^2^_p_ = .029; female targets (*M* = 4.44, *SD* = .76) were perceived as more attractive than males (*M* = 4.32, *SD* = .86), and a significant main effect of participant gender, *F*(1, 248) = 6.93, *p* = .009, η^2^_p_ = .027, such that female participants (*M* = 4.50, *SD* = .70) provided higher attractiveness ratings than males (*M* = 4.26, *SD* = .75). There was also a significant interaction between target and participant gender, *F*(1, 248) = 4.65, *p* = .032, η^2^_p_ = .018, such that male participants rated females (*M* = 4.37, *SD* = .77) as significantly more attractive than males (*M* = 4.15, *SD* = .94), *t*(125) = 2.95, *p* = .004, *d* = .26, 95% CI [.08, .44]. In contrast, female participants did not differ in their ratings of female (*M* = 4.51, *SD* = .75) and male targets (*M* = 4.49, *SD* = .75), *t*(123) = .52, *p* = .606, *d* = .05, 95% CI [-.13, .22]. The interaction between veracity and participant gender was not statistically significant, *F*(1, 248) = 2.17, *p* = .142, η^2^_p_ = .009, nor was the three-way interaction of veracity, target gender, and participant gender, *F*(1, 248) = 2.59, *p* = .109, η^2^_p_ = .010.

Again, we tested for mediation, using the same bootstrapping method from Studies 1-3, and found evidence to support that the multi-item measures of each warmth and openness were mechanisms through which veracity affected ratings of attractiveness [H3]. Warmth was a statistically significant mediator (indirect effect = 0.18, *SE* = 0.03, 95% CI [.11, .25]), as was openness (indirect effect = .11, *SE* = .03, 95% CI [.06, .17]). As in previous studies, warmth and openness were highly correlated (*r_truth_­* = .72, *r_lie_* = .73), and a composite immediacy variable was created. This immediacy construct was also a significant mediator of the relationship between veracity and attractiveness (indirect effect = .19, *SE* = .03, 95% CI [.12, .26]).

**Binary Measure of Attractiveness.** Finally, we analyzed participant responses on a binary measure of attractiveness, as an analogue to the binary measure of veracity. Here, when we use the term ‘accuracy’ we are referring to identifying a truth-teller as attractive and a lie-teller as unattractive on the binary attractive/unattractive measure. Overall, the binary attractiveness accuracy score (52%) was small, but significantly above chance (50%), *t*(249) = 5.20, *p* < .001, *d* = .33, 95% CI [.20, .46].

A 2 (veracity) x 2 (participant gender) x 2 (target gender) mixed ANOVA on percentage of accurate attractiveness judgments revealed a main effect of veracity, *F*(1, 248) = 97.61, *p* < .001, η^2^_p_ = .282. Truth-tellers (*M* = 67.47%; *SD* = 25.36) were more accurately labeled (as attractive) than lie-tellers (as unattractive; *M* = 37.48%; *SD* = 24.92). A main effect of target gender also emerged, *F*(1, 248) = 24.82, *p* < .001, η^2^_p_ = .091. Female targets (*M* = 54.75%; *SD* = 9.64) were more accurately identified than male targets (*M* = 50.20%; *SD* = 11.10). A main effect of participant gender, *F*(1, 248) = 6.05, *p* = .015, η^2^_p_ = .024, indicated that male participants (*M* = 53.64%; *SD* = 10.44) achieved greater accuracy on this measure than female participants (*M* = 51.31%; *SD* = 10.61). A veracity by participant gender interaction emerged, *F*(1, 248) = 6.05, *p* < .001, η^2^_p_ = .024. Male participants were more accurate at detecting truths (*M* = 62.57%; *SD* = 26.20) than lies (*M* = 44.71, *SD* = 25.14) on the binary attractiveness measure, *t*(125) = 4.10, *p* < .001, *d* = .37, 95% CI [.18, .55]. A similar pattern was present for female participants (truth-tellers: *M* = 72.38%, *SD* = 24.47; lie-tellers: *M* = 30.24%, *SD* = 24.70), but the effect size was larger, *t*(123) = 9.97, *p* < .001, *d* = .90, 95% CI [.67, 1.10]. A veracity by target gender interaction, *F*(1, 248) = 15.99, *p* < .001, η^2^_p_ = .061, was also present. For male targets, truth-tellers (*M* = 62.73%; *SD* = 29.25) were more accurately detected than lie-tellers (*M* = 37.67%, *SD* = 29.91), *t*(249) = 7.22, *p* < .001, *d* = .46, 95% CI [.33, .59]. The same was true for female targets (truth-tellers: *M* = 72.13%, *SD* = 28.48; lie-tellers: *M* = 37.40%, *SD* = 28.73), but the size of this effect was larger, *t*(249) = 10.23, *p* < .001, *d* = .65, 95% CI [.51, .78]. Finally, a target gender by participant gender interaction was statistically significant, *F*(1, 248) = 5.11, *p* = .025, η^2^_p_ = .020. Male participants were more accurate at detecting female (*M* = 56.94%, *SD* = 10.15) than male targets (*M* = 50.33%, *SD* = 11.71), *t*(125) = 4.81, *p* < .001, *d* = .43, 95% CI [.25, .61]. Female participants were also more accurate at detecting female (*M* = 52.55%, *SD* = 10.44) versus male targets (*M* = 50.07%, *SD* = 9.09), *t*(123) = 2.07, *p* = .040, *d* = .19, 95% CI [.01, .36], but this effect was smaller than observed for male participants. The three-way interaction between veracity, target gender, and participant gender was not significant, *p* = .609.

**Direct, Veracity Judgments**

**Continuous Measure of Veracity.** We first calculated mean ratings of veracity for truth-tellers and lie-tellers (collapsing across target gender). We found that truth-tellers were rated as significantly more truthful (*M* = 4.88, *SD* = 0.70) than lie-tellers (*M* = 4.45, *SD* = 0.70), *t*(250) = 11.97, *p* < 0.001, *d* = 0.76, 95% CI [.62, .90].

To provide a more complete analysis, including potential gender effects, we also conducted a 2 (veracity) x 2 (participant gender) x 2 (target gender) mixed ANOVA on mean ratings of the continuous veracity item. This analysis revealed a main effect of veracity, *F*(1, 248) = 142.99, *p* < .001, η^2^_p_ = .366. Truth-tellers (*M =* 4.88*; SD =* .70) were rated as more likely to be telling the truth than lie-tellers (*M =* 4.45*; SD =* .71). A main effect of target gender was also significant, *F*(1, 248) = 7.94, *p* = .005, η^2^_p_ = .031. Female targets (*M =* 4.73*; SD =* .73) were perceived as more likely to be telling the truth than male targets (*M =* 4.61*; SD =* .74). Additionally, there was a significant interaction between veracity and target gender, *F*(1, 248) = 15.65, *p* < .001, η^2^_p_ = .059. Participants rated female truth-tellers as more likely to be telling the truth (*M* = 4.88, *SD* = .83) than female lie-tellers (*M* = 4.58, *SD* = .80), *t*(249) = 6.31, *p* < .001, *d* = 0.40, 95% CI [.27, .53]*.* Participants also rated male truth-tellers as more likely to be telling the truth (*M* = 4.89, *SD* = .85) than male lie-tellers (*M* = 4.33, *SD* = .82), *t*(249) = 11.31, *p* < .001, *d* = 0.72, 95% CI [.58, .85], although this effect was greater than that observed for female targets*.* All other main effects and interactions were non-significant, *p*s > .118.

**Binary Measure of Veracity.** Most deception detection research uses a binary measure of veracity and calculates accuracy for lies and truths (Bond & DePaulo, 2006). Taking this approach, overall accuracy was at 54%, significantly above chance (50%), *t*(249) = 7.59, *p* < .001, *d* = 0.48, 95% CI [.35, .61]. This accuracy rate is consistent with meta-analytic estimates of deception detection accuracy (54%; Bond & DePaulo, 2006), but has an effect size considerably smaller than the continuous measure of veracity described above.

A 2 (veracity) x 2 (participant gender) x 2 (target gender) mixed ANOVA on percentage of accurate veracity judgments revealed a main effect of veracity, *F*(1, 248) = 228.819, *p* < .001, η^2^_p_ = .480. Truth-tellers (*M =* 70.06%*; SD =* 18.02) were detected more accurately than lie-tellers (*M =* 38.50%*; SD =* 19.45). A main effect of target gender was also significant, *F*(1, 248) = 8.11, *p* = .005, η^2^_p_ = .032. Female targets (*M =* 52.80%*; SD =* 12.33) were less accurately detected than male targets (*M =* 55.76%*; SD =* 11.92). Additionally, there was a significant interaction between veracity and target gender, *F*(1, 248) = 7.91, *p* = .005, η^2^_p_ = .031. Participants were more accurate at detecting female truth-tellers (*M* = 70.33%, *SD* = 21.25) than female lie-tellers (*M* = 35.27%, *SD* = 22.84), *t*(249) = 15.15, *p* < .001, *d* = 0.96, 95% CI [.81, 1.11]*.* Participants were also better at detecting male truth-tellers (*M* = 69.80%, *SD* = 22.85) versus male lie-tellers (*M* = 41.73%, *SD* = 23.72), *t*(249) = 11.09, *p* < .001, *d* = 0.70, 95% CI [.56, .84], although this effect was smaller than that observed for female targets*.* All other main effects and interactions were non-significant, *p*s > .400.

**Analysis Summary**

Using an ANOVA approach to data analysis, this generalized the truth attraction effect; participants who were made fully aware that some individuals would be lying also discriminated between lie-tellers and truth-tellers on a measure of attractiveness. Another finding from this pre-registered was that continuous measures produced much greater effect sizes than binary ones. This was true both for indirect, attractiveness ratings and direct ratings of veracity. Future researchers should carefully consider how they measure veracity judgments as the choice to use a continuous measure may afford greater sensitivity to find effects, despite the fact that prior research on lie-detection accuracy has primarily used binary measures (Bond & DePaulo, 2006).

**Supplemental Study 3**

This study replicates Supplemental Study 2. It is exactly the same as the study above except that we did not include binary measure of attractiveness and veracity—we used only continuous measures.

**Methods**

**Participants**

Two hundred participants completed the study through a cloud-based participant recruitment platform, using two separate postings, one for males and one for females, to recruit a gender balanced sample. Participants who failed attention checks or indicated that they had technical difficulties (e.g., video did not play) were excluded, leaving one hundred and eighty-six participants for analysis (48.9% identified as female); 72% White, 14% Black, 4.8% Latine, 6% Asian, .5% South Asian, 1.6% American Indian, and 1.1% identified as other or of mixed-race. The age of participants ranged from 19 to 73 years old (*M* = 35.99, *SD* = 9.71*)*. Lastly, 78% of participants identified as heterosexual. This study was not pre-registered, but deidentified data are available at: <https://osf.io/38tbv/?view_only=017a3e032b6e4d74889d282002b04733>

**Materials**

The same lie/truth stimuli used in Study 1 were used in here, except for one video which was changed from a female truth-teller to a female lie video to evenly balance the number of truth tellers and liars in the female videos. Participants watched all 24 videos, and the audio/speech was perceptible (i.e., not content-filtered).

**Procedures**

Just as in Supplemental Study 2 above, participants were told that they would be watching videos of people who each deny stealing $100 and that they would be answering questions on the targets of the videos. After reading those instructions, participants were presented all 24 videos in randomized order. After each video, participants rated the target on a number of traits: 1-item physical attractiveness rating (On a scale from 1 to 7, “how attractive do you think this person is as compared to all the people you have met in your life?—meaning, how attractive are they as a human—when comparing to others?”), 1-item deception rating (“On a scale from 1 to 7, please rate how much you think the person in the video is lying”), 5-item warmth measure (on a scale from 1 to 7, “Please rate how much you believe the word [caring, sincere, tolerant, likeable, good natured] applies to the person in the video you watched”) and an 8-item openness measure (“On a scale from 1 to 7, please rate how much you agree with the following statement: the person in the video [is original; comes up with new ideas; is curious about many different things; is ingenuous; is a deep thinker; has an active imagination; is inventive; values artistic, aesthetic experiences; prefers work that is routine; likes to reflect; plays with ideas”]. After watching all 24 videos participants answered demographic questions.

**Results**

**Indirect, Attraction Judgments**

Overall, truth-tellers were found to be more attractive (*M* = 4.89, *SD* = .85*)* than lie-tellers (*M* = 4.76, *SD* = .85), *t*(185) = 4.65*, p* <.001, *d* = .34. Splitting analyses by participant gender, males perceived truth-tellers to be more attractive (*M* = 4.88, *SD* = .80) than lie-tellers (*M* = 4.72, *SD* = .83), *t*(94) = 3.77, *p* < .001, *d* = .39*.* Specifically, when viewing female targets, males found female truth-tellers significantly more attractive (*M* = 5.08, *SD* = .83*)* than female lie-tellers (*M =* 4.73, *SD* = .91*), t*(94) = *6.15, p* < .001, *d* = .63*.* However, when male participants did not find male truth-tellers more attractive (*M* = 4.68*, SD* = .93) than male lie-tellers (*M* = 4.71*, SD* = .90*)*, *t*(94) = -.61, *p* = .54. Female participants perceived truth-tellers to be significantly more attractive (*M* = 4.91*, SD* = 0.90) than lie-tellers (*M* = 4.80, *SD* = .87), *t*(90)= 2.75, *p* = .007, *d =* .29. Specifically, females perceived female truth-tellers as significantly more attractive (*M* = 5.08, *SD* = .86) than female lie-tellers (*M* = 4.81, *SD* = .91), *t*(90) = 4.54, *p* < .001, *d* = .48. However, females did not find male truth-tellers more attractive (*M* = 4.74, *SD* = 1.02) than male lie-tellers (*M* = 4.79, *SD* = .99), *t*(90) = -.90, *p* = .368.

Warmth scores were calculated by taking the average of the 5 warmth items for each target, and then averaging the warmth composite across truth-tellers and lie-tellers separately. The same method was used to create openness scores for truth-tellers and lie-tellers. We used the same within-subjects bootstrap mediation, with 5,000 simulations, as in Studies 1-4. Ratings of targets’ warmth mediated the relation between attractiveness judgements and veracity (indirect effect = .14, SE = .03, 95% CI [.08, .19]). Ratings of targets’ openness also mediated the relation between attractiveness judgements and veracity (indirect effect = .14, SE = .03, 95% CI [.08, .19]).

As in Studies 1-3 warmth and openness were highly correlated (*r*_truth_ = .78, *r*_lie_ = .82), thus we created an immediacy variable for truth-tellers and lie-tellers by averaging the warmth and openness scores. Again, the immediacy construct mediated the relation between veracity and attractiveness (indirect effect = .16, SE = .03, 95% CI [.11, .22].

**Direct, Veracity Judgments**

Continuous veracity measures were calculated in a similar manner to attractiveness ratings, such that ratings were averaged to create a mean rating for truth-tellers and liars, separately. This rating was reversed-scored to have higher scores reflect perceived truth-telling, and lower scores reflect perceived lie-telling. Overall, participants rated truth-tellers as more truthful (*M* = 4.12, *SD* = 1.11) than lie-tellers (*M* = 3.92, *SD* = .98), *t*(185) = 4.78, *p* < .001, *d* = .35.

Splitting analyses by participant gender, male participants rated truth-tellers as more truthful (*M* = 4.05, *SD* = 1.06) than lie-tellers (*M* = 3.87, *SD* = .93), *t*(94) = 3.01, *p* = .003, *d* = .31*.* Specifically, males did not perceive female truth-tellers as more truthful (*M* = 4.07, *SD* = 1.16) than liars (*M* = 3.95, *SD* = 1.04), *t*(94) = 1.49, *p* = .138. However, males perceived male truth-tellers as more truthful (*M* = 4.04, *SD* = 1.10) than liars (*M* = 3.79, *SD* = .96), *t*(94) = 3.08, *p* = .002, *d* = .32. Female participants rated truth-tellers as more truthful (*M* = 4.18, *SD* = 1.16) than liars (*M* = 3.96, *SD* = 1.03), *t*(90) = 3.80, *p* < .001, *d* = .39. Specifically, females did not perceive female truth-tellers as more truthful (*M* = 4.20, *SD* = 1.26) than liars (*M* = 4.08, *SD* = 1.17), *t*(90) = 1.51, *p* = .134. However, females perceived male truth-tellers as more truthful (*M* = 4.16, *SD* = 1.20) than liars (*M* = 3.85, *SD* = .98), *t*(90) = 4.06, *p* < .001, *d* = .43.

*How Do Indirect, Attractiveness and Direct, Veracity Ratings Compare?*

We conducted a 2 (judgement type: attractiveness vs. veracity) x 2 (veracity: truth vs. lie) within-subjects ANOVA. There was a main effect of judgement type, *F*(1, 185) = 58.20, *p* < .001, *d* = 1.12, driven by attractiveness judgements being higher overall compared to direct deception judgements. There was also a main effect of target veracity, *F*(1, 185) = 34.17, *p* < .001, *d* = .859, as noted above. However, there was no interaction between judgement type and veracity, *F*(1, 185) = 2.29, *p* = .132, *d* = .220, which suggests that both judgements perform similarly in discriminating liars from truth-tellers.

**Supplemental Study 4**

The purpose of this study was to directly compare indirect, attractiveness and direct, veracity ratings. However, after conducting the study we discovered that the baseline attractiveness of the targets were not well balanced. Although this flaw limits conclusions that can be made from this study, we feel the data are potentially of use and test H1 and H2, finding the same effects as other studies without this flaw.

**Methods**

**Participants**

One hundred and eighty-two participants completed the study through a university in the north west. Participants who failed attention checks or had technical difficulties were excluded from analysis, leaving one hundred and sixty-four participants (66.4% identified as female); 78.7% white, 1.2% black, 7.9% Latinx, 8.5% Asian/Pacific Islander, and 3.7% other and/or of mixed race. The age ranged from 18 to 32 years old (*M* = 19.77, *SD* = 1.72); 85.9% of participants identified as heterosexual. This study was not pre-registered, but deidentified data are available at: <https://osf.io/38tbv/?view_only=017a3e032b6e4d74889d282002b04733>

**Materials**

Twenty-four high-stakes mock crime videos were used. The stimuli largely overlapped with those used in Study 4; however, the stimuli that differed meant that the baseline physical attractiveness of the truth-telling females was somewhat higher than the other groups—thus stacking the deck in the favor of Hypotheses 1 and 2. We did not discover this until after the study was completed—initial pilot-testing suggested the four cells (female-truth, male-truth, female-lie, and male-lie) were balanced on attractiveness; however, a second set of raters was collected post hoc and this set revealed the difference noted above. We report the data nonetheless.

**Procedures**

Participants were randomly assigned to rate targets with direct measures of veracity (truth/lie), or to rate targets with indirect measures (attractiveness). In the veracity condition participants were asked “How truthful is this person?” (on a continuous 7-point scale) and “Is this person lying or telling the truth?” (binary judgement) In the indirect condition participants were asked “How attracted are you to this person?” (on a continuous 7-point scale) and “Would you want to spend an evening getting to know this person?” (binary judgement). These questions were the only differences between conditions, and no other variables were measured for this study. In this study, participants only watched cross gender videos; males viewed female targets, females viewed male targets.

**Results**

*Were Truth-Tellers More Attractive Than Liars?*

Attractiveness scores for truth-tellers and liars were calculated similarly to the other studies reported in the main manuscript and in the SOM. Overall, participants perceived truth-tellers as more attractive (*M =* 2.32, *SD* = .90) than liars (*M* = 2.09, *SD* = .89), *t*(73) = 3.04, *p* = .003, *d* = .35. Males perceived female truth tellers (*M =* 2.73*, SD =* 1.09) as more attractive than female liars (*M =* 2.15*, SD =* 1.14), *t*(21) = 4.39, *p* < .001, *d* = .94. However, female perceivers did not find male truth-tellers (*M* = 2.14*, SD* = .75) more attractive than male liars (*M* = 2.07*, SD* = .78), *t*(51) = .89, *p* = .38.

*How Accurate were the Explicit Measures of Deception?*

Continuous veracity ratings were calculated similarly to attractiveness ratings. Using this measure, participants perceived truth-tellers as significantly more truthful (*M* = 4.41, *SD* = .74) than liars (*M* = 3.90, *SD* = .77), *t*(89) = 5.77, *p* < .001, *d* = .61. Males perceived female truth-tellers as more truthful (*M* = 4.30, *SD* = .62) than liars (*M* = 3.87, *SD* = .79), *t*(32) = 2.78, *p* = .009, *d* = .48. And, females perceived male truth-tellers as more truthful (*M* = 4.48, *SD* = .80) than liars (*M* = 3.91, *SD* = .76), *t*(56) 5.15, *p* < .001, *d* = .68.

When using a binary measure of veracity, participants performed slightly below chance, though not significantly, at 49%, *t*(89) = -.86, *p* = .393. Accuracy for detecting liars (46%) did not differ from chance, *t*(89) = -1.77, *p* = .079, nor did truth-detection accuracy (51%), *t*(89) = .56, *p* = .578. We also examined how often participants selected the ‘telling the truth’ versus ‘lying’ response option, finding that participants were more likely to report targets as telling the truth (*M* = 6.74, *SD* = 2.06*)* than lying (*M* = 5.26, *SD* = 2.06*), t*(89) = -3.42, *p* < .001, *d* = .36.

Splitting data by participant gender, male participants’ accuracy when examining female targets was slightly below chance at 45%, t(32) = -1.93, *p* = .062. Specifically, accuracy for discerning liars (41%) was below chance, *t*(32) = -2.41, *p* = .022, *d* = .42, while accuracy for discerning truth-tellers (49%) did not differ from chance, *t*(32) = -.24, *p* = .813. Female participants’ accuracy when judging male targets was at chance (50%), *t*(56) = .19, *p* = .849. Specifically, female participants’ accuracy for discerning liars (48%) and truth-tellers (53%) did not differ from chance, *t*(56) = -.61, *p* =.546 and *t*(56) = .86, *p* = .396, respectively. Overall, the SOM Study 2 supports H1 and H2, and replicates findings presented in the main manuscript.

1. All analyses presented below replicate when sample is restricted to individuals who identify as heterosexual. [↑](#footnote-ref-1)
2. Due to a survey error 24 participants were not shown a fertility prompt, leaving data from 221 participants for the fertility manipulation analysis; all participants are in other analyses that do not depend on the fertility manipulation. [↑](#footnote-ref-2)
3. Only participants identifying as either male or female were used in analyses including gender as a variable. [↑](#footnote-ref-3)
